# *Acinetobacter baumannii* in sheep, goat, and camel raw meat: virulence and antibiotic resistance pattern

**DOI:** 10.3934/microbiol.2019.3.272

**Published:** 2019-09-23

**Authors:** Neda Askari, Hassan Momtaz, Elahe Tajbakhsh

**Affiliations:** 1 Ph.D Student of Microbiology, Department of Microbiology, Shahrekord Branch, Islamic Azad University, Shahrekord, Iran; 2 Department of Microbiology, Shahrekord Branch, Islamic Azad University, Shahrekord, Iran

**Keywords:** *Acinetobacter baumannii*, antibiotic resistance pattern, Iran, ruminant meat, virulence factors

## Abstract

*Acinetobacter* genus belongs to a group of Gram-negative coccobacillus. These bacteria are isolated from human and animal origins. Antimicrobial agents play a vital role in treating infectious diseases in both humans and animals, and *Acinetobacter* in this regard is defined as an organism of low virulence. The current study aimed to evaluate antibiotic resistance properties and virulence factor genes in *Acinetobacter* *baumannii* strains isolated from raw animal meat samples. Fresh meat samples from 124 sheep, 162 goat, and 95 camels were randomly collected from Isfahan and Shahrekord cities in Iran. Most *A. baumannii* strains isolated from sheep meat samples represented *fimH* (82.35%), *aac(3)-IV* (78.43%), *sul1* (78.43%) and Integron Class I (96.07%) genes. Moreover, more than 50% of *A. baumannii* strains isolated from sheep samples were resistant to streptomycin (54.90%), gentamycin (74.50%), co-trimoxazole (70.58%), tetracycline (82.35%), and trimethoprim (62.74%). Current findings revealed significant association between the presence of *fimH, cnfI, afa/draBC, dfrA1*, *sulI*, *aac(3)-IV* genes in sheep samples. Furthermore, significant association was observed between *fimH, cnfI, sfa/focDE* and *dfrA1*genes in goat meat samples. In sheep meat samples, significant differences were identified in resistance to gentamicin, tetracycline, and co-trimoxazole in comparison with other antibiotics. Finally, there were statistically significant differences between the incidences of resistance to gentamicin, tetracycline, and co-trimoxazole in comparison with other antibiotics in all strains. In conclusion, the presence of virulence factors and antibiotic resistance in *A. baumannii* strains isolated from animal meat samples showed that animals should be considered as a potential reservoir of multidrug-resistant *A. baumannii*.

## 1. Introduction

*Acinetobacter baumannii* (*A. baumannii*) has been considered a major nosocomial pathogen, especially in intensive care units (ICUs), which can produce numerous infections such as septicemia, nosocomial meningitis, urinary tract infection, bacteremia, wound infection, infection of skin and soft tissue, and high-mortal pneumonia [1]. Interestingly, it can be identified in a range of food items, such as fruits, raw vegetables, milk, and dairy products [2]. There are few science reports of infections due to *A. baumannii* in animals and only a scarce number of studies have reported such cases [3].

Of this genus, *A. baumannii* strains revealed more resistant patterns than other species and often express a multi-drug resistant (MDR) phenotype. Consequently, during the past 30 years, strains of *A. baumannii* have obtained resistance against newly developed antimicrobial agents. This fact has become prevalent in some hospitals in the world and has been identified as a complicated nosocomial pathogen [4]. Antimicrobial agents are essential for treating infectious diseases in both humans and animals [5].

Based on the degree of antimicrobial resistance for *Acinetobacter* *spp.*, there are three different terms: multidrug resistant (MDR), extensive drug resistant (XDR), and pan-drug resistant (PDR). MDR *Acinetobacter* *spp*. refers to resistance pattern to a minimum of three classes of antimicrobial drugs such as penicillin and cephalosporin, fluoroquinolone, and aminoglycoside [6]. Another multidrug resistance definition belongs to the resistance pattern to more than two of the following five antibiotic drug classes: antipseudomonal cephalosporin (ceftazidime or cefepime), antipseudomonal carbapenem (imipenem or meropenem), ampicillin-sulbactam, fluoroquinolone (ciprofloxacin or levofloxacin), and aminoglycoside (gentamicin, tobramycin, or amikacin) [7].

*Acinetobacter* is defined as an organism of low virulence, which among the possible virulence factors, one can mention cell surface hydrophobicity, outer membrane proteins (OMPs), toxic slime polysaccharides, and verotoxins. Many factors like extracellular enzymes, cytotoxins and secreted vascular permeability are produced by *A. baumannii* that are involved in the pathogenesis and cause harm to host tissues particularly in respiratory tract infections [8].

The pathogenesis in bacteria can be due to the prevalence of virulence factors, which are involved in some performances such as colonizing on the epithelium, evading and inhibiting the host's immune response through biofilm formation, and obtaining nutrition from the host [9]. However, there is little information about the virulence factors in *A. baumannii* and identifying these factors can develop novel therapeutic alternatives for the control of clinically relevant pathogen [10].

Only few research studies have stimulated data regarding *A. baumannii* in veterinary medicine. The issue of emerging pathogen in veterinary medicine with a high potential for multidrug resistance and prevalence of *A. baumannii* is becoming alarmingly evident [10]. Actually, *Acinetobacter* *spp*. have been isolated from different animal sources such as birds, fish, and rainbow trout. Moreover, some chicken septicemia, mastitis and metrititis in cows, abortions in cattle, pigs and horses, keratoconjunctivitis in cattle, omphalitis in calves, ear infections in cats, and respiratory infections and balanoposthitis in horses have been identified [10].

Based on research by Vaneechoutte et al. [11], seven *A. baumannii* isolates were identified from jugular catheter tips placed in horses, but the organism was only indicated as responsible for local infection or colonization. Francey et al. [12] identified the clinical characteristics of several pets, which suffer from different *A. baumannii* infections such as urinary, respiratory, wound and bloodstream infections, reporting an overall attributable mortality of 47%.

Studies regarding the association of *A. baumannii* strains with foodborne illnesses are somewhat limited. Therefore, the present investigation was done to study the prevalence rate and phenotypic characterization of antibiotic resistance of the A. baumannii strains isolated from sheep, goat, and camel raw meat samples.

## 2. Material and methods

### 2.1. Study samples

A total of 124 sheep, 162 goat, and 95 camels fresh raw meat samples were randomly selected from 106 meat shops in Isfahan and Shahrekord. All samples were taken from the femur muscle of animal species. Thirty grams of meat were collected from each animal. The cross-sectional study was conducted from December 2015 to September 2016. It is notable that all the samples were approved (healthy) by the specialized veterinarians of Shahrekord Azad University.

### 2.2. Isolation and identification of A. baumannii

Specimens were collected by a laboratory technician, properly labeled, and transferred immediately to the microbiology laboratory. Each of these samples were streaked on blood agar (Merck, Germany) and MacConkey agar (Merck, Germany), and then incubated aerobically at 37 °C for 24 hours. Further, non-hemolytic, opaque and creamy colonies on blood agar and non-lactose fermenting colonies on MacConkey agar were sub-cultured on MacConkey agar and incubated for 24 hours at 37 °C to achieve pure colonies. The isolated organisms were identified based on colonial and microscopic characteristics and different biochemical tests according to standard laboratory methods. Stock cultures were conserved in both agar slant and 20% sterile buffered glycerin and were maintained at -70 °C [13].

A DNA Extraction Kit (Cinnagen, Iran) was used to extract genomic DNA from the bacterial isolates according to the manufacturer's instructions. To confirm and recognition of the isolates, conventional polymerase chain reaction (PCR) was performed by amplification of 16S–23S ribosomal DNA and the primer pairs shown in Table 1.

PCRs were carried out in 50 µL volume, the ingredients of which consisted of 5 µL of 10X PCR buffer, 2 mM of MgCl2, 150 µM of dNTPs mix, one unit of Taq DNA polemerase (Fermentas-Lithuania), 1 µM of reverse and forward primers, and 3 µL of template DNA (being the DNA of the isolates). PCR program was set to a cycle of 6 min at 94 °C, 30 repetitive cycles, of 95 °C for 60 s, 58 °C for 60 s, and 72 °C for 40 s, as well as a last cycle of 72 °C for 5 min, respectively (Table 3) [15]. (Tavakol 2018). All PCR reactions were performed in a thermocycler (Eppendrof Mastercycler 5330; Eppendorf-Nethel-Hinz GmbH, Hamburg, Germany), and products of PCR amplification were visualized by electrophoresis in 1.5% agaros gel. Ultimately, fragment amplifications with a size of 208 bp illustrated the presence of *A. baumannii* in isolated samples.

### 2.3. Antimicrobial susceptibility patterns

Susceptibility of antimicrobial agents was assessed by the Kirby–Bauer disk diffusion method using Mueller–Hinton agar (HiMedia Laboratories, Mumbai, India, MV1084), according to the Clinical and Laboratory Standards Institute guidelines. After incubating the inoculated plate aerobically at 37 °C for 18–24 h, the *A.baumannii* isolates' susceptibility to each antimicrobial agents were measured and the results were interpreted in accordance with interpretive criteria provided by CLSI (2017) [16]. The antibimicrobial agents in this investigation were trimethoprim (5 µg/disk); tetracycline (30 µg/disk); ceftazidime (30 µg/disk); cephalothin (30 µg/disk); co-trimoxazole (23.75/1.25 µg/disk); tobramycin (10 µg/disk); amikacin (30 u/disk); gentamicin (10 µg/disk); streptomycin (10 µg/disk); erythromycin (15 µg/disk); rafampicin (5 µg/disk); azithromycin (15 µg/disk); nitrofurantoin (300 µg/disk); chloramphenicol (30 µg/disk); mupirocin (30 µg/disk); imipenem (10 µg/disk); levofloxacin (5 µg/disk), and ciprofloxacin (5 µg/disk). For quality control purposes, the *A.* *baumannii* ATCC 19606 was used to determine antimicrobial susceptibility.

**Table 1.** Primers used for detection of virulence genes in *A. baumannii* [14].

Gene	Primer name	Primer Sequence (5′–3′)	Size of product (bp)
*afa/draBC*	afa1	GCTGGGCAGCAAACTGATAACTCTC	750
	afa2	CATCAAGCTGTTTGTTCGTCCGCCG	
*cnf1*	cnf1	AAGATGGAGTTTCCTATGCAGGAG	498
	cnf2	CATTCAGAGTCCTGCCCTCATTATT	
*cnf2*	cnf2a	AATCTAATTAAAGAGAAC	543
	cnf2b	CATGCTTTGTATATCTA	
*csgA*	M464	ACTCTGACTTGACTATTACC	200
	M465	AGATGCAGTCTGGTCAAC	
*cvaC*	ColV-CF	CACACACAAACGGGAGCTGTT	680
	ColV-CR	CTTCCCGCAGCATAGTTCCAT	
*fimH*	FimH F	TGCAGAACGGATAAGCCGTGG	508
	FimH R	GCAGTCACCTGCCCTCCGGTA	
*fyuA*	FyuA f	TGATTAACCCCGCGACGGGAA	880
	FyuA R	CGCAGTAGGCACGATGTTGTA	
*ibeA*	ibe10 F	AGGCAGGTGTGCGCCGCGTAC	170
	fibe10 R	TGGTGCTCCGGCAAACCATGC	
*iutA*	AerJ F	GGCTGGACATCATGGGAACTGG	300
	AerJ R	CGTCGGGAACGGGTAGAATCG	
*kpsMT* II	kpsII F	GCGCATTTGCTGATACTGTTG	272
	kpsII R	CATCCAGACGATAAGCATGAGCA	
*PAI*	RPAi F	GGACATCCTGTTACAGCGCGCA	930
	RPAi R	TCGCCACCAATCACAGCCGAAC	
*papC*	pap1	GACGGCTGTACTGCAGGGTGTGGCG	328
	pap2	ATATCCTTTCTGCAGGGATGCAATA	
*PapG* II, III	pGf	CTGTAATTACGGAAGTGATTTCTG	1070
	pGr	ACTATCCGGCTCCGGATAAACCAT	
*sfa/focDE*	sfa1	CTCCGGAGAACTGGGTGCATCTTAC	410
	sfa2	CGGAGGAGTAATTACAAACCTGGCA	
*traT*	TraT F	GGTGTGGTGCGATGAGCACAG	290
	TraT R	CACGGTTCAGCCATCCCTGAG	
*A. baumannii* detection	16S-23S	(F) CATTATCACGGTAATTAGTG	208
	ribosomal DNA	(R) AGAGCACTGTGCACTTAAG	

### 2.4. Virulence factors, antibiotic resistance and integrons genes detection

The virulence genes, antibiotic resistance coding genes, and integrons are presented in tables 1 and 2. PCR programs (temperature and volume) for detection of 16S–23S ribosomal DNA and all mentioned genes in *A. baumannii* are summarized in Table 3 [15]. The PCR amplified products (10µL) were subjected to electrophoresis in a 1.5% agaros gel (Fermentas, Germany) in 1X TBE buffer (Fermentas, Germany) at 80V for 30 minutes, stained with DNA Safe Stain (Cinnagen, Iran), which were subsequently examined under ultra violet illumination (Uvitec, England). In current study, in order to detect the molecular mass of PCR products the 100-bp ladder (Fermentas, Germany) was used as a standard factor, and to finalize the PCR results (confirm or reject), the PCR products of the primary positive samples were purified by a PCR product purification kit (Roche Applied Science, Germany) and sent to the Macrogen Co. (South Korea) for sequencing.

**Table 2.** Primers used for detection of antibiotic resistance genes in *A. baumannii* [17].

Gene	Primer Sequence (5′-3′)	Size of product (bp)
*aadA1*	(F) TATCCAGCTAAGCGCGAACT	447
	(R) ATTTGCCGACTACCTTGGTC	

*aac(3)-IV*	(F) CTTCAGGATGGCAAGTTGGT	286
	(R) TCATCTCGTTCTCCGCTCAT	

*sul1*	(F) TTCGGCATTCTGAATCTCAC	822
	(R) ATGATCTAACCCTCGGTCTC	

*blaSHV*	(F) TCGCCTGTGTATTATCTCCC	768
	(R) CGCAGATAAATCACCACAATG	

*CITM*	(F) TGGCCAGAACTGACAGGCAAA	462
	(R) TTTCTCCTGAACGTGGCTGGC	

*cat1*	(F) AGTTGCTCAATGTACCTATAACC	547
	(R) TTGTAATTCATTAAGCATTCTGCC	

*cmlA*	(F) CCGCCACGGTGTTGTTGTTATC	698
	(R) CACCTTGCCTGCCCATCATTAG	

*tet(A)*	(F) GGTTCACTCGAACGACGTCA	577
	(R) CTGTCCGACAAGTTGCATGA	

*tet(B)*	(F) CCTCAGCTTCTCAACGCGTG	634
	(R) GCACCTTGCTGATGACTCTT	

*dfrA1*	(F) GGAGTGCCAAAGGTGAACAGC	367
	(R) GAGGCGAAGTCTTGGGTAAAAAC	

*Qnr*	(F) GGGTATGGATATTATTGATAAAG	670
	(R) CTAATCCGGCAGCACTATTTA	

*Imp*	(F) GAATAGAATGGTTAACTCTC	188
	(R) CCAAACCACTAGGTTATC	

*Vim*	(F) GTTTGGTCGCATATCGCAAC	382
	(R) AATGCGCAGCACCAGGATAG	

*Sim*	(F) GTACAAGGGATTCGGCATCG	569
	(R) GTACAAGGGATTCGGCATCG	

*Oxa-51-like*	(F) TAATGCTTTGATCGGCCTTG	353
	(R) TGGATTGCACTTCATCTTGG	

*Oxa-23-like*	(F) GATCGGATTGGAGAACCAGA	501
	(R) ATTTCTGACCGCATTTCCAT	

*Oxa-24-like*	(F) GGTTAGTTGGCCCCCTTAAA	246
	(R) AGTTGAGCGAAAAGGGGATT	

*Oxa-58-like*	(F) AAGTATTGGGGCTTGTGCTG	599
	(R) CCCCTCTGCGCTCTACATAC	

*IntI*	F: CAG TGG ACA TAA GCC TGT TC	160
	R: CCC GAC GCA TAG ACT GTA	

*IntII*	F: TTG CGA GTA TCC ATA ACC TG	288
	R: TTA CCT GCA CTG GAT TAA GC	

*IntIII*	F: GCC TCC GGC AGC GAC TTT CAG	1041
	R: ACG GAT CTG CCA AAC CTG ACT	

**Table 3.** PCR conditions for virulence genes, antibiotic resistance genes and integrons detection in *A. baumannii*.

*draBC, cnf1, csgA, cvaC, iutA, fyuA*	1 cycle: 95 °C ------------ 4 min. 30 cycle: 95 °C ------------ 50 s 58 °C ------------ 60 s 72 °C ------------ 45 s 1 cycle: 72 °C ------------ 8 min	5 µL PCR buffer 10X 1.5 mM Mgcl2 200 µM dNTP (Fermentas) 0.5 µM of each primers F & R 1.25 U Taq DNA polymerase (Fermentas) 2.5 µL DNA template
*cnf2, kpsMT II, PAI, papC*	1 cycle: 94 °C ------------ 6 min. 34 cycle: 95 °C ------------ 50 s 58 °C ------------ 70 s 72 °C ------------ 55 s 1 cycle: 72 °C ------------ 10 min	5 µL PCR buffer 10X 2 mM Mgcl2 150 µM dNTP (Fermentas) 0.75 µM of each primers F & R 1.5 U Taq DNA polymerase (Fermentas) 3 µL DNA template
*fimH, ibeA, PapG II-III, sfa/focDE, traT*	1 cycle: 95 °C ------------ 4 min. 34 cycle: 94 °C ------------ 60 s 56 °C ------------ 45 s 72 °C ------------ 60 s 1 cycle: 72 °C ------------ 10 min	5 µL PCR buffer 10X 2 mM Mgcl2 200 µM dNTP (Fermentas) 0.5 µM of each primers F & R 1.5 U Taq DNA polymerase (Fermentas) 5 µL DNA template
*16S-23S ribosomal DNA*	1 cycle: 94 °C ------------ 6 min. 30 cycle: 95 °C ------------ 60 s 58 °C ------------ 60 s 72 °C ------------ 40 s 1 cycle: 72 °C ------------ 5 min	5 µL PCR buffer 10X 2mM Mgcl2 150 µM dNTP (Fermentas) 1 µM of each primers F & R 1 U Taq DNA polymerase (Fermentas) 3 µL DNA template
*aadA1, aac(3)-IV, sul1, blaSHV, CITM, cat1, cmlA, tet(a), tet(B), dfrA1, and qnr.*	1 cycle: 94 °C ------------ 6 min. 33 cycle: 95 °C ------------ 70 s 55 °C ------------ 65 s 72 °C ------------ 90 s 1 cycle: 72 °C ------------ 8 min	5 µL PCR buffer 10X 2 mM Mgcl2 150 µM dNTP (Fermentas) 0. 5 µM of each primers F & R 1.5 U Taq DNA polymerase (Fermentas) 2 µL DNA template
*imp, vim, and sim*	1 cycle: 95 °C ------------ 4 min. 30cycle: 95 °C ------------ 45 s 58 °C ------------ 60s 72 °C ------------ 40 s 1 cycle: 72 °C ------------ 5min	5 µL PCR buffer 10X 1.5 mM Mgcl2 100 µM dNTP (Fermentas) 1 µM of each primers F & R 1 U Taq DNA polymerase (Fermentas) 2.5 µL DNA template
*Oxa-23-like, Oxa-24-like, Oxa-51-like, Oxa-58-like)*	1 cycle: 94 °C ------------ 5 min. 32 cycle: 95 °C ------------ 50 s 60 °C ------------ 60 s 72 °C ------------ 70 s 1 cycle: 72 °C ------------ 10 min	5 µL PCR buffer 10X 2.5 mM Mgcl2 200 µM dNTP (Fermentas) 0.5 µM of each primers F & R 1.5 U Taq DNA polymerase (Fermentas) 2 µL DNA template
*Int I/II/III*	1 cycle: 94 °C ------------ 6 min. 35 cycle: 94 °C ------------ 60 s 56 °C ------------ 60 s 72 °C ------------ 45 s 1 cycle: 72 °C ------------ 6 min	5 µL PCR buffer 10X 1.5 mM Mgcl2 200 µM dNTP (Fermentas) 0.5 µM of each primers F & R 1 U Taq DNA polymerase (Fermentas) 2.5 µL DNA template

### 2.5. Statistical analysis

The obtained data were analyzed using IBM SPSS software version 18 and P values were calculated using Chi-square and Fisher's exact tests to identify statistically significant association between distribution of virulence genes, and antibiotic resistance properties of the *A. baumannii* strains isolated from sheep, goat, and camel meat samples. A P value < 0.05 was considered statistically significant.

## 3. Results

A total of 381 samples, 124 samples of sheep meat, 162 samples of goat meat and 95 samples from camel meat were obtained; of these, *A.* *baumannii* were isolated from 51 (41.12%) sheep meat samples, 19 (11.72%) from goat meat samples and 5 (2.26%) from camel meat samples (Table 4). Moreover, there is significant difference between sheep meat with other samples infected with *A. baumannii* (*P* = 0.025)

**Table 4.** Incidence of *A. baumannii* strains in sheep, goat and camel raw meat samples.

Samples	No. Samples	No. *A. baumannii*	Isolation rate
Sheep meat	124	51	41.12%
Goat meat	162	19	11.72%
Camel meat	95	5	2.26%
Total	381	75	19.68%

Interestingly, the highest virulence factor genes, which were detected in all strains isolated from samples were *fimH* (Table 5). Based on statistical analysis, there was significant association between the presence of *fimH, cnfI, afa/draBC* genes in this virulence factor gene with other genes in sheep samples (*P* = 0.031). In addition, there was significant association between *fimH, cnfI, sfa/focDE* genes with other genes in goat meat samples (*P* = 0.042), and strong association was observed between the presence of *fimH, cnfI, sfa/focDE, afa/draBC* virulence factor genes with others genes in camel isolated samples (*P* = 0.029). According to this table, it can be concluded that almost all virulence factor genes were represented in sheep samples except *fyuA.* For goat and camel samples, the presence of *PAI* and *papGIII* genes was not detected.

Table 6 demonstrates the frequency of antibiotic resistance genes in sheep, goat, and camel meat samples, which revealed that none of the three meat sample groups represented the *qnr* gene. The highest and lowest antibiotic resistance genes were *aac(3)-IV*, *sul1* and *sim* in sheep meat sample with resistance values of 80.39%, 80.39% and 1.96%, respectively. Moreover, in the studied samples, all genes represented except *qnr*. Statistical analysis revealed significant association between the presence of *dfrA1*, *sulI*, *aac(3)-IV* genes in sheep isolated samples (*P* = 0.019); in goat samples, strong association between *dfrA1* gene (*P* = 0.021) were observed, and in all sample groups, *dfrA1, sulI, aac(3)-IV* genes showed significant difference in comparison to other antibiotic resistance genes (*P* = 0.025).

Integron genes are presented in Table 7 and it can be concluded that the most frequent gene in all sample groups was the Integron Class I, but Integron Class III was not found in goat and camel positive samples. Overall, 85.33% of isolated *A. baumannii* strains represented Class I Integron genes. Statistics confirmed significant association between the presence of Integron Class I in sheep, goat and camel isolated samples (*P* = 0.019, 0.032 and 0.041, respectively); moreover, the presence of Integron Class I revealed significant difference in comparison to other Integron genes (*P* = 0.029).

Antimicrobial susceptibility tests conducted using the Kirby-Bauer test revealed that more than 50% of *A. baumannii* stains isolated from sheep samples were resistant to streptomycin, gentamycin, co-trimoxazole, tetracycline, and trimethoprim (Table 8). The highest resistance was observed in *A. baumannii* stains isolated from goat and camel meat samples belong to trimetoprim.

Statistical analysis showed significant difference between resistance to gentamicin, tetracycline, and co-trimoxazole in comparison with other antibiotics in isolated strains of sheep meat samples (*P* = 0.032). Overall, there were statistically significant differences amongst the incidences of resistance to gentamicin, tetracycline, and co-trimoxazole in comparison with other antibiotics in all strains (*P* = 0.026).

**Table 5.** Frequency of virulence factor genes in *A.* *baumannii* strains isolated from sheep, goat and camel meat samples.

Sample/frequency	*fimH*	*fyuA*	*iutA*	*cvaC*	*csgA*	*Cnf2*	*Cnf1*	*afa/draBC*
Sheep/51	42	-	17	12	11	17	24	27
Goat/19	14	7	7	3	7	3	11	6
Camel/5	3	-	-	1	-	-	1	1
Sample/frequency	*traT*	*Sfa/focDE*	*PapG III*	*PapG II*	*papC*	*ibeA*	*PAI*	*KpsMT II*
Sheep/51	2	27	3	11	8	10	2	13
Goat/19	3	4	-	3	6	3	-	4
Camel/5	1	1	-	-	1	1	-	-

**Table 6.** Frequency of antibiotic resistance genes in *A.* *baumannii* strains isolated from sheep, goat and camel meat samples.

Sample/frequency	*aadA1*	*aac(3)-IV*	*sul1*	*bla_SHV_*	*bla_CITM_*	*tetA*	*tetB*	*dfrA1*	*qnr*
Sheep/51	28	40	40	24	21	18	26	36	-
Goat/19	9	9	11	10	10	7	5	15	-
Camel/5	1	2	2	2	1	2	-	3	-
Sample/frequency	*vim*	*sim*	*Imp*	*cat1*	*cmlA*	*Oxa-51-like*	*Oxa-23-like*	*Oxa-24-like*	*Oxa-58-like*
Sheep/51	3	1	2	2	5	5	3	6	4
Goat/19	2	-	1	3	-	2	1	-	3
Camel/5	-	-	-	-	2	1	-	1	2

**Table 7.** Frequency of Integron genes in *A.* *baumannii* strains isolated from sheep, goat and camel meat samples.

Class I	Class II	Class III	Sample/frequency
49	16	1	Sheep/51
11	6	-	Goat/19
4	1	-	Camel/5

**Table 8.** Antibiotic resistance pattern in *A.* *baumannii* strains isolated from sheep, goat and camel meat samples.

Sample/frequency	streptomycin	gentamicin	amikacin	tobramycin	co-trimoxazole	cephalotin	ceftazidime	tetracycline	trimetoprim
Sheep/51	28	38	21	20	36	19	16	42	32
Goat/19	7	8	10	9	11	10	12	11	14
Camel/5	-	2	-	1	2	-	-	2	3
Sample/frequency	ciprofloxacin	lovofloxacin	imipenem	meropenem	cloramphenicol	nitrofurantoin	azithromycin	rifampin	erythromycin
Sheep/51	12	7	5	7	6	7	4	6	22
Goat/19	7	3	-	2	3	4	2	4	8
Camel/5	1	-	-	-	2	1	-	-	1

## 4 Discussion

In literatures, *Acinetobacter* are denoted as a heterogeneous group of organisms that are found almost everywhere, frequently distributed in the environment. The isolated strains of this species have frequently originated from animals including birds, fish, and rainbow trout [6]. Researchers have rarely assessed the infections due to *A. baumannii* in animals. Among previous findings, we could mention the study on *A. baumannii* isolates from different animals including ducks, pigeons, chickens, donkeys, rabbits, pets (cats and dogs), mules, livestock (cattle, sheep, goats, pigs), horses, lice and arthropods, which outbreaks in human medicine have been reported in several years [18],[19].

In the present study, antibiotic resistance patterns and virulence factors, and antibiotic resistance genes and Integron genes were assessed, which most sheep meat samples with *A. baumannii* represented *fimH*, *aac(3)-IV*, *sul1* and Integron Class I genes. Nonetheless, among all isolated stains, all genes except *qnr* were represented. Eventually, more than 50% of *A. baumannii* stains isolated from sheep meat samples were resistant to streptomycin, gentamycin, co-trimoxazole, tetracycline, and trimetoprim. The most resistance pattern in *A. baumannii* stains isolated from goat and camel meat samples belongs to trimetoprim. Some research findings in other research groups revealed the same result as our investigation; however, some research findings contradicted with our findings. For example, Francey et al. showed the clinical characteristics of several pets with various *A. baumannii* infections such as urinary, respiratory, wound and bloodstream infections. Their findings revealed an overall attributable mortality of 47% [12]. A research discovery presented that all 16 *A. baumannii* isolates from food-producing animals were sensitive to imipenem, meropenem, and ciprofloxacin and piperacillin/tazobactam; however, those samples were resistant to ceftazidime [20]. In another survey on 16 *A. baumannii* isolates, the outcomes proved 100% sensitivity to carbapenems, gentamicin, ciprofloxacin, and piperacillin/tazobactam; nonetheless, those samples were resistant to amoxicillin, cefradine, trimethoprim, and chloramphenicol [21].

In a research project by Rafei et al. (2015) [22], a total of 73 water samples, 51 soil samples, 37 raw cow milk samples, 50 cow meat samples, 7 raw cheese samples, and 379 animal samples were analyzed to detect the presence of *A. baumannii*. *A. baumannii* was found in 6.9% of water samples, 2.7% of milk samples, 8.0% of meat samples, 14.3% of cheese samples, and 7.7% of animal samples. All isolates in this survey presented a susceptible phenotype to most of the antibiotics tested with seldom findings regarding carbapenemase-encoding genes, except one that harbored a *bla*OXA-143 gene. This study verified that animals could be a potential reservoir for *A. baumannii* and dissemination of new emerging carbapenemases.

Gram-Negative Bacteria (GNB) that possessed *A. baumannii* with some other species had been assessed and isolated from mastitic milk samples of dairy cattle. Findings revealed that half of the GNB isolates were resistant to 5 or more of the 12 tested antimicrobial agents [23]. Some antibiotic agents were evaluated in 57 *A. baumannii* bulk tank milk (BTM) isolated samples; their findings showed resistance patterns to cefepime, imipenem, meropenem, ciprofloxacin, levofloxacin, and colistin [24].

## 5. Conclusion

In the current survey, it can be inferred that although most of the isolated samples were resistant against antibiotics, more than half of *A. baumannii* stains isolated from sheep samples were resistant against streptomycin, gentamycin, co-trimoxazole, tetracycline, and trimetoprim. For goat and camel meat positive samples, the highest resistance belongs to trimetoprim. The most represented antibiotic resistance genes in sheep meat samples were *fimH*, *aac(3)-IV*, *sul1* and Integron Class I. Interestingly, the *dfrA1* gene has been represented in most samples in all three sample groups; however, none of the isolated stains revealed the *qnr* gene. Entirely, almost 90% of each isolated cluster were represented with Integron Class I. *A.* *baumannii* were isolated mainly from sheep, goat and camel meat samples in Iran; hence, animals should be considered as a potential reservoir of multidrug-resistant *A. baumannii*. Due to various factors involved in the infection in different animal species, further studies are crucial for gaining a better understanding of the origins of this infection in humans. Some limitation factors noted in the current study were restricted sample population, retrospective data analysis, unreliable species identification methods, or unique reports of accidental observations.
